# Intergenerational associations between parental low-carbon concern and early adolescents’ low-carbon consumption behaviors: a moderated mediation model

**DOI:** 10.3389/fpsyg.2026.1836570

**Published:** 2026-09-03

**Authors:** Jian Li, Qingyi Fang, Xueting Zhang

**Affiliations:** 1 School of Business, Anhui University, Hefei, China; 2 School of Business Administration, Anhui University of Finance and Economics, Bengbu, China

**Keywords:** ecological socialization, intergenerational transmission, low-carbon concern, low-carbon consumption behavior, parent-adolescent dyads, parent–child relationship quality

## Abstract

Low-carbon consumption on the demand side is essential for achieving long-term cleaner production and systemic emission reduction. While existing research has predominantly focused on adult consumers and individual-level determinants, considerably less is known about how low-carbon consumption behaviors develop during early adolescence and how family processes are involved in their early formation. Drawing on an ecological socialization perspective, this study examines whether parental low-carbon concern is associated with early adolescents’ low-carbon consumption behaviors through adolescents’ own low-carbon concern. Using multi-informant data from 399 matched parent-adolescent dyads in China, we tested a moderated mediation model. Parental low-carbon concern was positively associated with early adolescents’ habitual and purchasing low-carbon consumption through adolescents’ own low-carbon concern. Adolescent-perceived parent–child relationship quality strengthened this indirect association, whereas adolescents’ environmental self-identity showed an unexpected negative moderation effect. These findings provide a differentiated account of family environmental socialization in low-carbon consumption, while the unexpected moderation by environmental self-identity requires further investigation.

## 1 Introduction

Global warming stands as one of the most pressing environmental challenges of the 21st century, exerting profound effects on both ecosystems and human societies. According to the International Energy Agency, global energy-related carbon dioxide (CO₂) emissions reached an unprecedented 37.80 billion tons in 2024, a 0.80% increase largely attributed to rising coal use in China, India, and Southeast Asia (IEA, 2025). China alone generated 12.60 billion tons of carbon dioxide equivalent emissions, with household consumption accounting for more than half (Meng et al., 2023). Despite growing urgency, low-carbon consumption remains inadequately understood and insufficiently internalized among Chinese consumers (Di et al., 2024; Ma and Chen, 2025; Jiang et al., 2020). Anthropogenic greenhouse gas emissions continue to be the dominant force behind this ongoing crisis (Kennedy et al., 2009; Wang C. et al., 2023; Wang J. et al., 2023).

Achieving carbon neutrality ultimately depends on transforming consumption patterns toward sustainability. A key challenge is encouraging consumers to consider low-carbon products and services in their everyday decisions (Gong et al., 2022; Liu et al., 2022; Ren et al., 2024). Prior studies have identified a range of determinants of low-carbon consumption, which can be grouped into three broad categories. The first includes individual-level factors such as attitudes, values, and self-identity (Jiang et al., 2019; Chen and Li, 2019; Mi et al., 2019). The second involves sociodemographic characteristics such as income and education (Karytsas and Theodoropoulou, 2014; Yang et al., 2016; Cheng et al., 2023). The third encompasses contextual influences such as policy frameworks, media communication, and prevailing social norms (Du et al., 2017; Ding et al., 2025; Wang C. et al., 2023).

Most existing studies, however, have focused on adults, while early adolescents have received comparatively less attention. Adolescence is a formative period during which personal values and consumption habits continue to develop, and patterns established during this stage may persist into adulthood (Lusinga and De Groot, 2019). Adult consumption patterns, by contrast, are often more established and resistant to change (Gong et al., 2022). The United Nations Sustainable Development Goals also recognize youth as a central force in driving environmental transformation (UNESCO, 2017). Early adolescents may therefore represent a relevant population for understanding the development of sustainable consumption orientations (Testa et al., 2021; Geraci et al., 2024). Between the ages of 10 and 15, adolescents undergo substantial psychological development, while their capacity to independently evaluate complex environmental consequences continues to evolve (Valkenburg et al., 2017; Gong et al., 2022; Wang J. et al., 2023). Parental guidance consequently remains salient in the development of their environmental concerns and consumption orientations.

Existing studies have primarily drawn on the theory of planned behavior and value-belief-norm theory to explain the cognitive, normative, and motivational antecedents of low-carbon consumption behavior (Ajzen, 1991; Stern et al., 1999; Ding et al., 2018; Zhang et al., 2022; Ren et al., 2024; Ma and Chen, 2025). Although these frameworks offer valuable explanations of attitudes, norms, values, and behavioral intentions, they give comparatively less attention to developmental context and family socialization processes (Jiang et al., 2019; Chen and Li, 2019; Mi et al., 2019; Ding et al., 2025). This limitation is particularly relevant during adolescence, when environmental beliefs and consumption patterns are still developing and parental guidance remains salient. A fuller understanding of early adolescents’ low-carbon concerns and consumption behaviors therefore requires complementing individual-level frameworks with a developmental and ecological socialization perspective.

The ecological socialization perspective views parents as early and influential agents in adolescents’ environmental development because family interactions provide important contexts for observing, discussing, and interpreting environmental values and practices (Gentina and Muratore, 2012; Gentina and Singh, 2015; Erickson et al., 2016). Early adolescents occupy a transitional stage between dependence and autonomy but may remain responsive to parental guidance in relatively complex domains such as environmental consumption (Erickson et al., 2016; Gong et al., 2022). Socialization refers to the process through which individuals encounter and internalize values, motivations, and practices within a sociocultural context (Gentina and Muratore, 2012). Consistent with this perspective, research has examined how parental environmental orientations are associated with children’s and adolescents’ pro-environmental values and behaviors (Lusinga and De Groot, 2019; Davies et al., 2020; Pearce et al., 2020; Essiz and Mandrik, 2022; Gong et al., 2022). However, general environmental consciousness does not necessarily correspond to concern about consumption-related carbon emissions. The intergenerational association of carbon-specific concern with early adolescents’ consumption behavior therefore remains insufficiently understood.

To address this issue, the present study integrates ecological socialization and intergenerational transmission perspectives to examine whether parental low-carbon concern is associated with early adolescents’ low-carbon consumption through adolescents’ own low-carbon concern (Gentina and Muratore, 2012; Li et al., 2023). Given adolescents’ developing cognitive capacities and limited autonomy in consumption decisions, parents play a pivotal role in shaping their environmental concerns and behavioral tendencies. Elucidating this transmission mechanism provides a theoretical basis for designing effective low-carbon education and behavioral intervention strategies that cultivate environmental responsibility among the next generation.

This study investigates adolescents’ low-carbon concerns as a mediating mechanism and highlights the moderating role of parent–child relationship in the intergenerational transmission of low-carbon concerns. Prior research indicates that adolescents who perceive closer and more supportive parent–child relationships may be more receptive to parental communication and value internalization (Perez-Brena et al., 2014; Gong et al., 2022; Wang J. et al., 2023). Environmental self-identity refers to the extent to which individuals view environmentally responsible behavior as part of who they are and has been associated with personal norms and pro-environmental behavior (Li et al., 2023; van der Werff et al., 2013, 2014; Yang and Laroche, 2011). We therefore examine whether these relational and identity-based conditions are associated with variation in the link between parental and adolescent low-carbon concern.

Using multi-informant data from 399 matched parent-adolescent dyads in China, this study tests a moderated mediation model. The results indicate that parental low-carbon concern is positively associated with early adolescents’ habitual and purchasing low-carbon consumption through adolescents’ own low-carbon concern. Parent–child relationship is associated with a stronger indirect association. Environmental self-identity also conditions this process, although the observed negative moderation is contrary to H3 and is therefore treated as an exploratory finding.

This study makes three focused theoretical contributions. First, it extends family environmental socialization research from broad environmental orientations to consumption-related low-carbon concern. Building on ecological socialization theory, it explains how parental concern about carbon emissions is associated with adolescents’ own concern and low-carbon consumption (Gentina and Muratore, 2012; Gentina and Singh, 2015; Gong et al., 2022). Second, it identifies adolescents’ low-carbon concern as a psychological pathway linking parental concern to both habitual and purchasing low-carbon consumption. This process-based account advances prior work that has mainly documented parent–child similarities in environmental attitudes and behaviors, including recycling, energy conservation, and green purchasing (Matthies et al., 2012; Hansen and Jacobsen, 2020; Wallis and Klöckner, 2020). Third, it examines parent–child relationship quality and environmental self-identity as relational and identity-based boundary conditions. A closer relationship strengthens the indirect association, consistent with family communication research (Koerner and Fitzpatrick, 2002). The negative moderation by environmental self-identity was contrary to our prediction and is therefore treated as an exploratory finding (van der Werff et al., 2013).

The remainder of this paper is organized as follows. The next section reviews relevant literature and develops the hypotheses. Subsequent sections describe the research methods, present the empirical results, and discuss their theoretical and practical implications.

## 2 Literature review

### 2.1 Ecological socialization theory and intergenerational transmission

Ecological socialization theory provides the central theoretical foundation for understanding how adolescents acquire environmental attitudes, values, and behaviors. It posits that parents serve as early and influential socialization agents because they shape the meanings, norms, and practices that adolescents encounter in everyday family life (Gentina and Muratore, 2012; Gentina and Singh, 2015). Within this perspective, family environmental socialization refers to the relational process through which adolescents observe, interpret, negotiate, and internalize environmental meanings. Intergenerational transmission represents a possible outcome of this process, reflected in the extent to which parental environmental orientations become associated with adolescents’ own concerns and behaviors (Gentina and Muratore, 2012; Meeusen, 2014).

Recent integrative reviews show that family environmental socialization operates through several mutually reinforcing channels, including parental modeling, environmental communication, shared practices, and family norms (Hosany et al., 2022; Liu and Green, 2024; Denault et al., 2024; Papa et al., 2025). Jia and Yu (2021) organize these processes around parental action, communication, and engagement. Parental action provides visible examples of environmentally responsible behavior. Communication conveys information, expectations, and moral meaning. Shared engagement enables adolescents to enact these values through routine activities such as energy conservation, waste reduction, recycling, and sustainable purchasing. Together, these processes can transform parental environmental orientations into personally meaningful concerns and behavioral tendencies among adolescents.

Intergenerational transmission, however, is neither automatic nor uniform. Empirical findings on parent-adolescent similarity remain mixed (Collado et al., 2019; Liu and Kaida, 2024; see Table 1). Meeusen (2014), for example, found that both maternal and paternal environmental concerns were positively associated with adolescents’ environmental concern. Other studies reported broad similarity but weaker environmental values among adolescents than among their parents (Grønhøj and Thøgersen, 2009; Casaló and Escario, 2016; Giancola et al., 2024). Some studies found limited evidence of successful transmission (Cipriani et al., 2013). These inconsistencies suggest that parental environmental orientations do not simply reproduce themselves in the next generation.

**Table 1:** Representative literature on the intergenerational transmission of pro-environmental attitudes and behaviors.

Published Year	Author	Method	Sample details	Major findings	Support the intergenerational transmission of pro-environmental attitudes and behaviors or not
2024	Liu and Kaida	Online survey matched data (parents and children)	815 parent–child pairs (children ages 9–18), Japan and China	There is a significant positive correlation between environmental attitudes, psychological barriers, and the behavior of parents and children.	Supported
2024	Giancola et al.	Cross-sectional survey matching data (parents and children)	229 triads of children (Mage = 8.54 years), fathers, and mothers, Italian	General moral judgments (e.g., moral transgressions, social conventions, and personal choices) were not found to significantly moderate the intergenerational association of pro-environmental behaviors.	Intergenerational association supported; moderating effect not supported
2023	Kong and Jia	Cross-sectional survey matched data (parents and adolescents)	396 adolescents (Mage = 10.82 years), 396 parents (Mage = 39.01 years), China	The environmental knowledge of both parents and children significantly contributes to their pro-environmental behaviors.	Supported
2022	Gong et al.	Cross-sectional survey matching data (parents and adolescents)	*N1* = 722, *N2* = 477 Matched parents and adolescents (10–15 years old), China	Parents’ green consumption values are positively associated with those of early adolescents, with environmentally responsible consumption behaviors mediating this relationship; the strength of this mediation varies with the quality of the parent–child relationship.	Supported
2022	Aruta and Paceño	Cross-sectional survey matching data (parents and adolescents)	437 parent-adolescent dyads, Philippines	The more parents attach importance to green consumption, the stronger their children will be in terms of social responsibility. This further leads to a greater tendency to form a green purchasing attitude or intention.	Supported
2022	Essiz and Mandrik	Cross-sectional survey matching data (mothers and college-age daughters)	146 mothers and college-age daughter (18–30 years old) dyads, Turkey	Mothers and daughters exhibit significant intergenerational similarities in sustainable consumption attitudes and behaviors. Moreover, daughters’ attitudes and behaviors in some cases exert a reverse influence on their mothers.	Supported
2020	Hansen and Jacobsen	Unbalanced panel data (Adults and their parents)	128,472 adults and parents, Denmark	There is a significant positive association between adults’ energy consumption patterns and those of their mothers, with this relationship being more pronounced among low-income adults.	Supported
2019	Collado et al.	Cross-sectional survey matching data (adolescents–mum–best friend triads)	N1 = 330 adolescents aged 12–19, N2 = 152 adolescents–mum–best friend triads, Spanish	Parental and peer descriptive and injunctive norms directly influence adolescents’ pro-environmental behaviors and also exert indirect effects through the development of personal norms.	Supported
2016	Casaló and Escario	Cross-sectional survey Matched data (Parents and Adolescents)	95,008, adolescents (15 years old) 16 countries and regions (Bulgaria, Colombia, Denmark, Germany, Hong Kong etc.)	Parents’ environmental concern tends to be passed on to their children, with mothers having a particularly strong influence—especially on daughters.	Supported More strongly for girls
2014	Meeusen	Secondary data (Parent Child Socialization Study)	Parents and offspring (3,426 adolescents, 15 years old), Dutch	The intergenerational transmission of parents’ environmental concern to their children, with particular emphasis on the mediating role of family communication patterns.	Supported
2013	Cipriani et al.	Experiment Matched data (Parents and Children)	76 (38 children and 38 parents) Washington, DC.	There was insufficient evidence to support the transmission of pro-social values from parents to their children.	Not supported
2012	Matthies et al.	Cross-sectional survey Matched data (Parents & Children)	206 Parent–child dyads (8–10 years old), German	While parents’ prescriptive norms influence children’s recycling behavior indirectly via their subjective norms, their descriptive norms show no significant effect. Conversely, in the context of reuse behavior, parents’ descriptive norms exert a significant influence, whereas prescriptive norms do not.	Partially supported
2009	Grønhøj and Thøgersen	Cross-sectional survey Matched data (Parents and Children)	601 families; Adolescents (75% above 15), With father and mother Denmark	There is a positive relationship between parents’ and adolescents’ pro-environmental commitment, which contains values, attitudes and behaviors. Adolescents show lower environmental concerns than parents.	Partially supported

Intergenerational parallels also vary across behavioral domains. They have been observed in energy conservation, green purchasing, recycling, and other forms of pro-environmental behavior (Matthies et al., 2012; Hansen and Jacobsen, 2020; Wallis and Klöckner, 2020; Aruta and Paceño, 2022; Gong et al., 2022). Yet their strength may depend on behavioral visibility, habituality, personal cost, and moral salience. Relational quality, cultural norms, and individual identity may further determine whether parental orientations are accepted and internalized (Essiz and Mandrik, 2022; Li et al., 2023; Giancola et al., 2024).

Ecological socialization theory also recognizes that adolescents are not passive recipients of parental influence. As autonomy and identity develop, they increasingly evaluate parental messages against their personal values, peer norms, and emerging self-concepts (Thomaes et al., 2023; Denault et al., 2024). Family environmental socialization may therefore involve acceptance, selective internalization, negotiation, resistance, or reverse influence. Its effectiveness depends not only on what parents model or communicate, but also on the relational climate in which socialization occurs and the extent to which adolescents have already formed an environmental identity.

Existing research thus establishes ecological socialization as an important foundation for adolescents’ environmental development. It also shows that parents contribute through modeling, communication, shared practices, and value transmission. Three questions remain unresolved. First, most studies examine broad environmental attitudes or general pro-environmental behavior, leaving the intergenerational transmission of consumption-related low-carbon concern less understood. Second, limited research has specified the psychological process through which parental low-carbon concern becomes associated with adolescents’ own concern and, subsequently, with distinct forms of low-carbon consumption. Third, the effectiveness of this process may depend on both adolescents’ receptiveness to parental influence and their emerging psychological autonomy. We address these questions by examining adolescents’ low-carbon concern as a mediating mechanism and parent–child relationship quality and environmental self-identity as relational and identity-based boundary conditions.

### 2.2 Low-carbon consumption behavior

Amid escalating environmental crises, low-carbon consumption has gained traction among academics and policymakers alike (Jiang et al., 2019; Mi et al., 2019; Xu et al., 2021). Low-carbon consumption behavior refers to consumer practices aimed at minimizing carbon emissions, including purchasing low-carbon consumption behavior (PLCB) and habitual low-carbon consumption behavior (HLCB) (Ding et al., 2018; Jiang et al., 2019). HLCB and PLCB represent conceptually distinct forms of low-carbon consumption. HLCB involves routine and relatively automatic practices (e.g., switching off lights, conserving water), whereas PLCB involves more deliberate and resource-dependent decisions (e.g., buying energy-efficient appliances, purchasing organic or locally produced food) (Barr et al., 2005; Mi et al., 2019; Cheng et al., 2023). We therefore examine both outcomes separately. However, because existing theory does not provide a sufficiently clear basis for predicting that the proposed indirect or moderated mediation process should be stronger for one behavioral domain than the other, no comparative directional hypothesis is advanced. Unlike general consumption, low-carbon consumption often requires individuals to trade off personal convenience or cost for long-term collective benefits. Parental influence is thus especially salient, as adolescents may lack the cognitive maturity to fully appreciate long-term consequences (Mi et al., 2019).

Prior research has identified diverse determinants of low-carbon consumption behavior, including psychological traits (e.g., attitudes, values, identity), sociodemographic variables, and situational contexts such as media and policy interventions (Ding et al., 2018; Ma and Chen, 2025; Wang C. et al., 2022). Psychological variables, particularly attitudes, subjective norms, and low-carbon awareness grounded in the theory of planned behavior, have played a central role in prior research (Jiang et al., 2019; Chen and Li, 2019; Gunarathne et al., 2020; Wu et al., 2023). Likewise, environmental values and personal norms derived from the value-belief-norm theory have also received extensive empirical support (Chen et al., 2014; Ding et al., 2025). Beyond internal cognition, contextual influences such as social norms, media exposure, policy cues, and social interactions play a significant role in shaping low-carbon behavior (Mi et al., 2019; Cheng et al., 2021; Ren et al., 2024). Other relevant constructs include ecological personality, environmental self-identity, and self-efficacy (Wei et al., 2016; Yue et al., 2021). These psychological and contextual factors interact in complex ways to shape consumption behaviors (Dai and Hu, 2022). The theory of planned behavior (Ajzen, 1991) and the value-belief-norm theory (Stern et al., 1999) remain dominant frameworks. Although these frameworks explain important individual cognitive and motivational determinants, they give comparatively less attention to developmental context and family socialization processes.

Existing literature has largely overlooked early adolescents, focusing instead on adult consumption patterns (Ding et al., 2018; Jiang et al., 2019; Chen and Li, 2019; Wu et al., 2023; Wollmar et al., 2024). Yet early adolescence is a formative period for value development and behavior formation. This study addresses this gap by examining how parental low-carbon concerns shape adolescents’ low-carbon concerns and behavior, contributing to a developmental understanding of low-carbon engagement.

## 3 Hypothesis development

### 3.1 Intergenerational transmission of parents’ low-carbon concerns

According to ecological socialization theory, parents serve as critical agents in transmitting environmental attitudes and values to their children, particularly during early adolescence (Gentina and Muratore, 2012; Essiz and Mandrik, 2022). Within the family, adolescents develop environmental orientations by observing parental behavior, discussing environmental issues, participating in shared practices, and receiving parental reinforcement (Singh et al., 2020; Wang J. et al., 2023). Parental low-carbon concern may therefore provide an important issue-specific cue through which adolescents learn how consumption relates to carbon emissions.

Low-carbon concern is conceptually related to, but narrower than, general environmental concern. In this study, low-carbon concern refers to the degree of cognitive attention and affective concern that individuals direct toward the carbon-emission consequences of their consumption activities and choices. Parental and adolescent low-carbon concern therefore represent the same construct assessed from each informant’s own perspective. Environmental concern captures broad cognitive and affective evaluations of environmental degradation, including pollution, biodiversity loss, resource depletion, and climate change (Nath et al., 2013; Singh et al., 2020). Low-carbon concern focuses specifically on carbon emissions and their consequences in consumption contexts. It captures the extent to which consumers attend to and care about the carbon implications of their consumption choices. It therefore represents a domain-specific manifestation of environmental concern rather than an entirely independent construct (Pagiaslis and Krontalis, 2014; Thøgersen, 2021; Wang et al., 2021).

Low-carbon concern also differs from adjacent environmental constructs. Environmental values reflect broad and relatively stable beliefs about the relationship between humans and nature (Stern et al., 1999; van der Werff et al., 2013). Pro-environmental attitudes refer to favorable or unfavorable evaluations of environmentally responsible actions or goals (Ajzen, 1991; Chen et al., 2014). Environmental knowledge or ecological awareness primarily concerns the recognition and understanding of environmental problems and their consequences (Pagiaslis and Krontalis, 2014; Wang et al., 2021). Low-carbon concern, by contrast, centers on cognitive and affective concern about consumption-related carbon emissions (Pagiaslis and Krontalis, 2014; Thøgersen, 2021). It does not itself denote environmental knowledge, a general value orientation, or an intention to act. This narrower focus makes it particularly relevant to habitual and purchasing forms of low-carbon consumption (Mi et al., 2019; Cheng et al., 2023).

Adolescents may internalize parental low-carbon concern through behavioral modeling, environmental communication, shared routines, and affective alignment. Parental behavior provides observable examples of how carbon considerations enter everyday consumption. Communication explains why these practices matter. Joint participation embeds low-carbon meanings in activities such as energy conservation, waste reduction, recycling, and sustainable purchasing (Liga et al., 2020; Jia and Yu, 2021; Tian et al., 2023). Emotional closeness may further increase adolescents’ receptiveness to parental values and expectations (Zhou and Hu, 2021). Consistent with this reasoning, Casaló and Escario (2016) found that both paternal and maternal environmental concerns were positively associated with adolescents’ environmental concerns.

Adolescents’ own low-carbon concern may, in turn, connect parental orientations with consumption behavior. Environmental concern is consistently associated with sustainable purchasing and green consumption (Abeliotis et al., 2010; Pagiaslis and Krontalis, 2014; Maduku, 2024). Research on climate-friendly consumption likewise identifies environmental concern, awareness, and perceived carbon consequences as relevant psychological antecedents of low-carbon action, although contextual barriers may prevent concern from translating uniformly into behavior (Thøgersen, 2021; Wang et al., 2021). Adolescents who attend more closely to the carbon consequences of consumption should therefore be more likely to adopt both routine low-carbon practices and deliberate low-carbon purchasing choices.

Taken together, ecological socialization theory suggests an indirect association in which parental low-carbon concern is linked to adolescents’ low-carbon consumption behaviors through adolescents’ own low-carbon concern. We therefore propose:

*H1*: Parental low-carbon concern is positively associated with early adolescents’ low-carbon consumption behaviors through adolescents’ own low-carbon concern.

### 3.2 The moderating effect of parent–child relationships

Parents serve as crucial agents in shaping their children’s early socialization, not only as primary caregivers but also as key influencers in the formation of their children’s values (Sjamsir et al., 2024). Through daily interactions, educational practices, and behavioral modeling, parents exert a subtle yet profound influence on the development of their children’s values (Lee, 2021). The intergenerational transmission of green consumption values has been found to be strongly influenced by the quality of the parent–child relationship. Specifically, the closer the parent–child relationship, the smoother the transmission of these values (Gong et al., 2022). Similarly, studies have shown that the intergenerational transmission of privacy concerns is also moderated by the parent–child relationship (Wang J. et al., 2023). Thus, the parent–child relationship plays a vital role in emotional, behavioral, and social development.

A positive parent–child relationship has been shown to promote prosocial behaviors and support healthy psychological development in children, while a poor relationship may hinder these developmental processes (Crockett et al., 2007). The parent–child relationships can be conceptualized in terms of the degree of closeness and attachment between parents and children (River et al., 2022; Zhou et al., 2023). A close and supportive parent–child relationship provides a solid foundation for open communication, emotional support, and understanding, especially during times of uncertainty or challenge (Crockett et al., 2007). In the context of low-carbon consumption, this relationship becomes particularly significant. When parents demonstrate care, encouragement, and support for their children’s low-carbon concerns within a warm and positive relationship, children are more likely to accept their parents’ guidance and internalize appropriate low-carbon values and behaviors.

However, when adolescents perceive the parent–child relationship as distant or strained, the association between parental low-carbon concern and adolescents’ own concern may be weaker. In such circumstances, adolescents may be less receptive to parental guidance or may interpret parental low-carbon messages as externally imposed expectations, potentially leading to superficial compliance or resistance (Crockett et al., 2007). Based on this reasoning, we propose that adolescent-perceived parent–child relationship quality conditions the indirect association between parental low-carbon concern and early adolescents’ low-carbon consumption behaviors through early adolescents’ own low-carbon concern.

*H2*: Parent–child relationship moderates the indirect association between parental low-carbon concern and early adolescents’ low-carbon consumption behaviors through adolescents’ own low-carbon concern. Specifically, the association between parental and adolescent low-carbon concern is stronger when adolescents perceive a closer parent–child relationship.

### 3.3 The moderating effect of environmental self-identity

The effectiveness of parental influence often hinges on children’s internal motivations and belief systems (Wu and Chao, 2011; Wang J. et al., 2023). Engaging in low-carbon behaviors often entails personal sacrifices, including additional time, financial expenditure, or reduced convenience. Individuals with a strong environmental self-identity are more likely to act consistently with that identity, even when doing so incurs personal costs.

Environmental self-identity reflects an individual’s self-perception as an environmentally conscious person (van der Werff and Steg, 2016). It facilitates the internalization of environmental values into one’s self-concept, thereby reinforcing sustainable behavioral patterns. As a relatively stable psychological construct, environmental self-identity has been shown to be a stronger predictor of pro-environmental behavior than environmental attitudes alone (Giancola et al., 2024). Research indicates that individuals with stronger environmental self-identity are more likely to engage in environmentally responsible behavior due to internal moral commitment (Whitmarsh et al., 2011; van der Werff et al., 2014). This self-perception transforms environmental actions into expressions of personal identity and moral responsibility (van der Werff et al., 2014). In adolescents, whose values are still being formed, environmental self-identity serves as a crucial mechanism linking biospheric values to sustained low-carbon behaviors (Jia and Liang, 2025).

Environmental self-identity may also shape how adolescents process parental low-carbon messages. Parental concerns that are consistent with adolescents’ environmental self-concept may be perceived as more self-relevant and receive greater cognitive and motivational attention. Adolescents with a stronger environmental self-identity may therefore be more receptive to identity-consistent parental messages and more likely to integrate these messages into their own low-carbon concern (van der Werff et al., 2013, 2014; Giancola et al., 2024). By contrast, adolescents with a weaker environmental self-identity may perceive parental expectations as more externally imposed and may be less likely to internalize them. Based on this identity-congruence reasoning, we propose:

*H3*: Environmental self-identity moderates the indirect association between parental low-carbon concern and early adolescents’ low-carbon consumption behaviors through early adolescents’ own low-carbon concern. Specifically, the association between parental and early adolescent low-carbon concern is stronger at higher levels of environmental self-identity.

**Figure 1:**
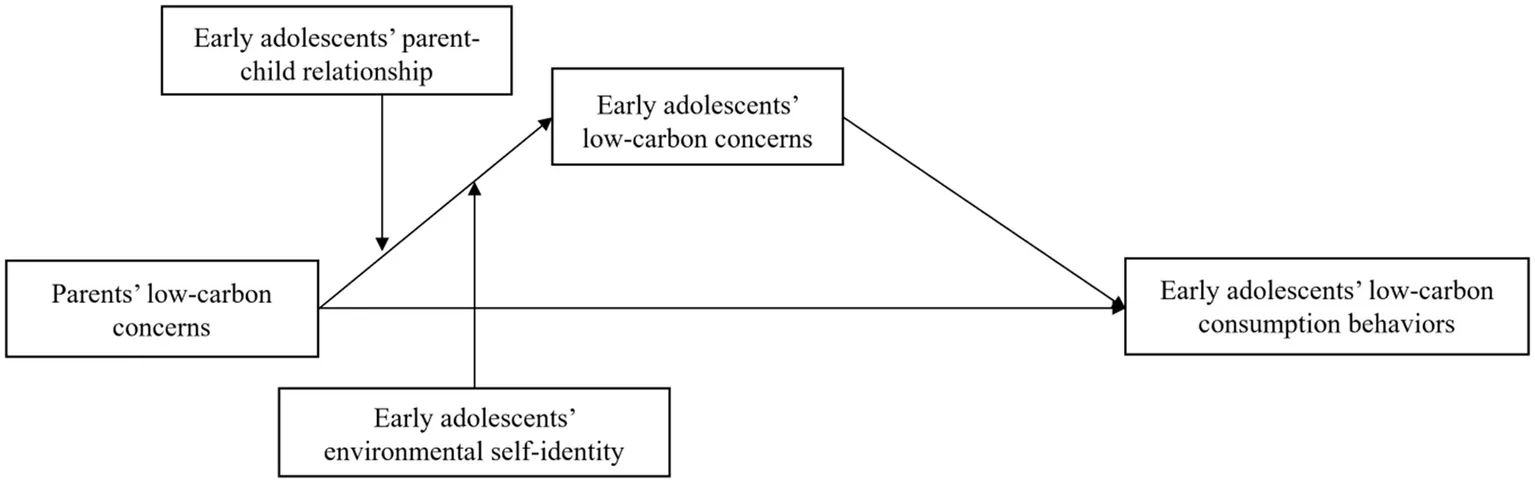
Conceptual model of the hypothesized moderated mediation relationships.

The research hypothesis model of this paper is shown in Figure 1.

## 4 Methods

### 4.1 Participants and procedures

To test the proposed research model, we used a matched parent-adolescent multi-informant design within Chinese families. Parents reported their own low-carbon concern, whereas adolescents reported their low-carbon concern, parent–child relationship, environmental self-identity, and low-carbon consumption behaviors. Participants were recruited from five middle schools located in central and eastern China. Two sets of questionnaires were prepared, sealed in envelopes, and assigned unique identifiers to facilitate anonymous matching of responses within families. All measurement scales employed in this study were adapted from well-established instruments validated in prior research. A rigorous translation and back-translation procedure was followed to ensure linguistic and conceptual equivalence between the original English scales and their Chinese versions.

The study deliberately focused on early adolescents enrolled in middle schools. Early adolescence was selected because environmental values and consumption habits remain under development, while parents continue to provide important guidance in complex consumption decisions (Valkenburg et al., 2017; Gong et al., 2022). Adolescents around age 18 were not included because they generally possess greater cognitive and consumption autonomy and may experience different patterns of parental influence. Including both early and late adolescents could therefore introduce developmental heterogeneity beyond the scope of the present study.

With the assistance of their class teachers, a total of 635 students and one of their parents were invited to participate in this study. Each student delivered the sealed envelope to a parent, which included a cover letter (outlining the research purpose, informed consent, and confidentiality assurance), demographic questions, and the low-carbon concern scale. After completing the survey, parents were instructed to reseal the envelope and return it to their child. The following day, homeroom teachers collected all responses and submitted them to the research team. One month later, students whose parents had completed the initial survey and consented to their children’s participation were invited to complete the adolescent portion of the questionnaire during class. The student questionnaire included the research purpose, informed assent, and confidentiality form, as well as items measuring demographics, low-carbon concerns, low-carbon consumption behavior, environmental self-identity, and the parent–child relationship. The one-month interval was designed to reduce cross-informant response contamination and the influence of immediate parent-adolescent discussions about the study. Participation was entirely voluntary, and all responses were de-identified and matched only through non-identifying codes. As an incentive, participants received a small gift valued at approximately USD 2.00.

A total of 399 matched parent-adolescent dyads were retained for analysis. Responses were excluded because of nonresponse, incomplete data, or unsuccessful parent-adolescent matching. Before data collection, written informed consent was obtained from each participating parent, who also provided permission for the adolescent’s participation, and written informed assent was obtained from each adolescent.

Table 2 presents the demographic characteristics of the study sample. Of the 399 adolescent participants, 192 were female (48.12%) and 207 male (51.88%), aged 10 to 15 years (*M* = 12.77, *SD* = 0.82). The majority were aged 12 or 13 (*n* = 321, 80.45%). Each family nominated one parent to complete the parent survey. The final valid sample comprised 168 fathers (42.11%) and 231 mothers (57.89%), aged 30 to 58 years (M = 40.92, SD = 4.31), with most (*n* = 228, 57.14%) between 40 and 49 years. Moreover, 17.0% of parents held a college or university degree or higher. The majority of families (61.40%) reported a disposable household income of ¥50,000–¥150,000. Regarding family composition, 298 households (74.69%) had two or more children, whereas 101 (25.31%) were single-child families.

**Table 2:** Participants’ demographic information.

Variables	Frequency	Percentage
Parents’ gender		
Male	168	42.11
Female	231	57.89
Children’s gender		
Male	207	51.88
Female	192	48.12
Parents’ age		
30–39	156	39.10
40–49	228	57.14
50–60	15	3.76
Children’s age		
10–11	18	4.51
12–13	321	80.45
14–15	60	15.04
Parents’ education level		
High school and below	256	64.16
Junior college	75	18.80
Bachelor’s degree	60	15.04
Master’s degree or above	8	2.00
Disposable household income (RMB)		
< 50,000	85	21.30
50,000-100,000	159	39.85
100,000-150,000	86	21.55
150,000-250,000	40	10.03
> 250,000	29	7.27
Family structure		
One child	101	25.31
Two children	174	43.61
Three children or more	124	31.08
Children’s grades		
First year of junior high school	216	54.14
Second year of junior high school	183	45.86

### 4.2 Measurement

#### 4.2.1 Parents and early adolescents’ low-carbon concerns

Adapted from Pagiaslis and Krontalis (2014), a three-item scale was used to assess low-carbon concern among both parents and adolescents. The items were: “How concerned are you about low-carbon consumption?”, “How concerned are you about pollution associated with carbon emissions?”, and “How concerned are you about carbon emissions when making purchases?” Responses were recorded on a five-point Likert scale ranging from 1 (not at all concerned) to 5 (extremely concerned). The scale demonstrated good internal consistency for both parents (Cronbach’s α = 0.86) and adolescents (Cronbach’s α = 0.83).

To assess whether the construct was measured comparably across the two informants, we conducted cross-informant measurement invariance tests using a paired CFA framework. The configural model showed an acceptable fit, *χ^2^*_(8)_ = 18.24, CFI = 0.993, TLI = 0.986, RMSEA = 0.057, and SRMR = 0.017. Constraining the corresponding factor loadings to equality did not reduce model fit, χ^2^_(10)_ = 19.25, CFI = 0.993, TLI = 0.990, RMSEA = 0.048, and SRMR = 0.020. The changes in fit indices were negligible, ΔCFI = 0.000, ΔRMSEA = 0.009, and ΔSRMR = 0.003, supporting metric invariance. Full scalar invariance was not supported. We therefore released the equality constraint on the intercept of the second item. The resulting partial scalar model showed a good fit, *χ^2^*_(11)_ = 22.30, CFI = 0.992, TLI = 0.989, RMSEA = 0.051, and SRMR = 0.021. Relative to the metric model, the changes were minimal, ΔCFI = 0.001, ΔRMSEA = 0.003, and ΔSRMR = 0.001. These results support configural, metric, and partial scalar invariance, indicating that the scale captured low-carbon concern in a broadly comparable manner across parents and adolescents.

#### 4.2.2 Early adolescents’ low-carbon consumption behavior

Low-carbon consumption behavior (LCB) was assessed using a two-dimensional framework adapted from Mi et al. (2019) and Cheng et al. (2023). This framework distinguishes between two types of behavior: purchasing low-carbon consumption behavior (PLCB) and habitual low-carbon consumption behavior (HLCB). PLCB refers to deliberate, resource-dependent actions, such as “When buying products, I consider their environmental impact.” HLCB reflects routine, everyday practices that minimize environmental impact, such as “I try to reduce the use of disposable products.” All items were rated on a five-point Likert scale ranging from 1 (strongly disagree) to 5 (strongly agree). Adolescents completed the scale as a self-report measure. The subscales showed acceptable internal consistency: PLCB (Cronbach’s α = 0.80) and HLCB (Cronbach’s α = 0.86).

#### 4.2.3 Early adolescents’ parent–child relationship

Parent–child relationship was assessed from the adolescent’s perspective using four items adapted from Resnick et al. (1997): “I am close to my mother/father,” “I feel that my mother/father cares about me,” “I am satisfied with my relationship with my mother/father,” and “I feel loved and needed by my family.” The scale captured adolescents’ perceptions of closeness, support, and emotional connection with the participating parent (Cronbach’s α = 0.94).

#### 4.2.4 Early adolescents’ environmental self-identity

Adapted from the study by van der Werff and Steg (2016), we used four items to measure the early adolescents’ environmental self-identity, including: “Acting environmentally friendly is an important part of who I am”, “I am the type of person who acts environmentally friendly”, “I see myself as an environmentally friendly person”, “My family and friends consider me to be an environmentally friendly person” (Cronbach’s α = 0.89).

#### 4.2.5 Control variables

Prior studies on low-carbon consumption behavior have identified several demographic characteristics—such as gender, education, and income—as important influencing factors (Ding et al., 2018). Accordingly, we controlled for three covariates in our analysis. First, we included the gender of the responding parent, with males coded as 0 and females as 1. Second, we controlled for annual household income, categorized as an ordinal variable. Third, we included the parent’s educational attainment, also measured as an ordinal variable. These controls help isolate the effects of the core psychological and relational constructs examined in this study.

## 5 Results

### 5.1 Descriptive statistics

Table 3 reports the means, standard deviations, and Pearson correlation coefficients for all study variables. The highest intercorrelation among the independent variables is 0.61, which falls well below the conventional multicollinearity threshold of 0.70 (Zhang et al., 2024), suggesting no severe bivariate overlap. Multicollinearity was further assessed using VIF diagnostics in the regression models. Moreover, parents’ low-carbon concerns were significantly and positively correlated with early adolescents’ habitual low-carbon consumption behavior (*r* = 0.47, *p* < 0.001), purchasing low-carbon consumption behavior (*r* = 0.46, *p* < 0.001), and early adolescents’ own low-carbon concerns (*r* = 0.57, *p* < 0.001). Similarly, early adolescents’ low-carbon concerns were positively associated with both habitual (*r* = 0.43, *p* < 0.001) and purchasing low-carbon consumption behavior (*r* = 0.45, *p* < 0.001). These significant correlations provide preliminary support for the proposed research hypotheses.

**Table 3:** Descriptive statistics, correlations, and discriminant validity.

Variables	M	SD	1	2	3	4	5	6	7	8
1. H_HLCB	4.32	0.60	**0.75**							
2. H_PLCB	4.20	0.67	0.56^***^	**0.76**						
3. F_LCA	4.22	0.73	0.47^***^	0.46^***^	**0.83**					
4. H_LCA	4.04	0.71	0.43^***^	0.45^***^	0.57^***^	**0.79**				
5. H_PCR	3.90	0.98	0.14^**^	0.12^*^	0.11^*^	0.36^***^	**0.89**			
6. H_ESI	3.72	0.82	0.23^***^	0.22^***^	0.30^***^	0.61^***^	0.13^**^	**0.81**		
7. Parents’ gender	0.58	0.49	−0.05	−0.01	−0.04	−0.01	−0.06	0.05		
8. Parents’ educational level	1.55	0.82	0.12^*^	0.01	0.04	0.03	0.01	−0.05	0.07	
9. Disposable household income	2.44	1.19	0.02	0.05	0.03	0.11^*^	0.01	0.04	0.06	0.34^***^

M = Mean; SD = Standard Deviation; H_HLCB, Early adolescents’ habitual low-carbon consumption behavior; H_PLCB, Early adolescents’ purchasing low-carbon consumption behavior; F_LCA, Parental low-carbon concerns; H_LCA, Early adolescents’ low-carbon concerns; H_PCR, Parent–child relationship; H_ESI, Environmental self-identity. The square roots of the average variance extracted (AVE) values are presented on the diagonal in boldface. ^*^p < 0.05, ^**^p < 0.01, ^***^p < 0.001.

To examine potential demographic differences, independent-samples t-tests were conducted by adolescent gender and participating-parent gender. Male adolescents reported higher low-carbon concern than female adolescents (*M_male_* = 4.21, *SD* = 0.56; *M_female_* = 3.85, *SD* = 0.80; *t* = −5.17, *p* < 0.001, *d* = 0.52) and higher environmental self-identity (*M_Male_* = 3.90, SD = 0.64; *M_female_* = 3.52, SD = 0.95; *t* = −4.69, *p* < 0.001, *d* = 0.48). Male adolescents also reported slightly higher parent–child relationship quality (*t* = −2.22, *p* = 0.02, *d* = 0.22). No significant gender differences were found for HLCB or PLCB. Parental low-carbon concern did not differ significantly between fathers and mothers (*t* = 0.88, *p* = 0.37, *d* = 0.09).

### 5.2 Common method bias test

To assess the potential impact of common method variance (CMV), we employed both Harman’s single-factor test and the unmeasured latent common method factor (ULCMF) approach (Bagozzi and Yi, 1990). Results from an exploratory factor analysis showed that the first unrotated principal component accounted for 38.13% of the total variance, which is well below the conventional threshold of 50%, suggesting that CMV is unlikely to pose a serious threat. Furthermore, results from confirmatory factor analysis (CFA) (see Table 4) indicated that the proposed six-factor model provided a good fit to the data (*χ^2^/df* = 1.85, *CFI* = 0.98, *NFI* = 0.94, *TLI* = 0.97, *IFI* = 0.98, *RMSEA* = 0.04) and fit significantly better than the single-factor model (Δχ^2^ = 2940.02, Δ*df* = 15, *p* < 0.001). In addition, comparisons between the six-factor model and the ULCMF model revealed only negligible differences in fit indices (ΔCFI < 0.01, ΔTLI = 0.01, ΔRMSEA < 0.01), further suggesting that no dominant common method factor was evident. However, because adolescents self-reported the mediator, moderators, and behavioral outcomes, these statistical diagnostics cannot fully rule out common-source variance.

**Table 4:** Fit indices for the measurement models.

Model	*χ^2^*	*df*	CFI	TLI	NFI	IFI	RMSEA
Six-factor model	358.93	194	0.98	0.97	0.94	0.98	0.04
Single-factor model	3298.95	209	0.43	0.37	0.42	0.44	0.19
Common method factor model with ULCMF	265.19	166	0.98	0.98	0.95	0.98	0.04

ULCMF refers to an unmeasured latent common method factor.

### 5.3 Reliability and validity tests

Tables 3, 5 report the reliability and validity results for the six constructs included in the CFA. Cronbach’s *α* values ranged from 0.80 to 0.94, indicating satisfactory internal consistency. Composite reliability values ranged from 0.80 to 0.94, and average variance extracted values ranged from 0.57 to 0.80, exceeding the recommended thresholds of 0.70 and 0.50, respectively (Hair et al., 2006). Table 5 further reports the standardized loadings of each of the 22 measurement items. The loadings ranged from 0.69 to 0.91 and were all above 0.50, providing additional support for convergent validity.

**Table 5:** Reliability and convergent validity of the constructs.

Construct	Items	Standardized loadings	CA	CR	AVE
Parents’ low-carbon concern	f_LCA1	0.88	0.86	0.87	0.68
	f_LCA2	0.83			
	f_LCA3	0.76			
Adolescents’ low-carbon concern	h_LCA1	0.77	0.83	0.83	0.62
	h_LCA2	0.82			
	h_LCA3	0.77			
Habitual low-carbon consumption behavior	h_HLCB1	0.72	0.86	0.87	0.57
	h_HLCB2	0.74			
	h_HLCB3	0.69			
	h_HLCB4	0.80			
	h_HLCB5	0.79			
Purchasing low-carbon consumption behavior	h_PLCB1	0.75	0.80	0.80	0.58
	h_PLCB2	0.72			
	h_PLCB3	0.81			
Parent–child relationship	h_PCR1	0.91	0.94	0.94	0.80
	h_PCR2	0.88			
	h_PCR3	0.89			
	h_PCR4	0.89			
Environmental self-identity	h_ESI1	0.88	0.89	0.88	0.66
	h_ESI2	0.84			
	h_ESI3	0.77			
	h_ESI4	0.74			

CR, Composite Reliability; CA, Cronbach’s α; AVE, Average Variance Extracted.

Discriminant validity was assessed using the Fornell–Larcker criterion (Fornell and Larcker, 1981). Specifically, the square root of each construct’s average variance extracted (AVE), ranging from 0.75 to 0.89, exceeded its highest correlation with any other construct, thereby confirming satisfactory discriminant validity. In addition, results from the confirmatory factor analysis (CFA) (see Table 4) indicated that the proposed six-factor model demonstrated a good fit to the data. Moreover, it fit the data significantly better than the one-factor model (*Δχ^2^* = 2940.02, *Δdf* = 15, *p* < 0.001), thereby providing strong support for the discriminant validity of the measurement model. Taken together, these results provide robust evidence of the reliability and construct validity of the measurement instruments employed in this study.

### 5.4 Hypothesis testing

Each matched parent-adolescent dyad was represented by one analytic record. The hypothesized model was directional and multi-informant: parental low-carbon concern was reported by parents, whereas the mediator, moderators, and outcomes were reported by early adolescents. Because the study did not propose reciprocal actor-partner effects or measure parallel outcomes for both dyad members, PROCESS was used to estimate the proposed mediation and first-stage moderated mediation effects. We used the SPSS PROCESS macro to test the proposed mediation and moderated mediation models within a regression-based conditional process framework (Hayes, 2017; Preacher et al., 2007). PROCESS Model 4 examined the indirect association between parental low-carbon concern and early adolescents’ low-carbon consumption behavior through early adolescents’ low-carbon concern. Model 7 tested parent–child relationship quality and environmental self-identity separately as first-stage moderators. Model 9 then estimated the complete conditional process model by including both moderators simultaneously on the path from parental to adolescent low-carbon concern. All continuous predictors were mean-centered before the interaction terms were constructed. We estimated the conditional indirect effects and indices of moderated mediation using 5,000 bootstrap samples and bias-corrected 95% confidence intervals. To assess multicollinearity, we reproduced the relevant PROCESS equations using equivalent OLS regressions that included the same mean-centered predictors, interaction terms, and control variables. As reported in Tables 6, 7, the VIF values ranged from 1.00 to 1.49, indicating that multicollinearity was unlikely to account for the estimated interaction effects.

**Table 6:** Regression results for the mediation and moderated mediation models (Equations 1–5).

Variables	Equation 1		Equation 2		Equation 3		Equation 4		Equation 5	
	H_HLCB		H_LCA		H_HLCB		H_LCA		H_LCA	
	*B*	*SE*	*B*	*SE*	*B*	*SE*	*B*	*SE*	*B*	*SE*
Constant	2.64^***^	0.16	1.69^***^	0.18	3.43^***^	0.18	3.84^***^	0.07	3.93^***^	0.06
Parent’s gender	−0.04	0.06	0.01	0.05	−0.03	0.05	0.02	0.05	−0.03	0.04
Disposable household income	−0.02	0.03	0.06^*^	0.03	−0.03	0.02	0.07^**^	0.02	0.05^**^	0.02
Parents’ educational level	0.09^*^	0.04	−0.02	0.04	0.10	0.03	−0.01	0.03	0.01	0.03
F_LCA	0.38^***^	0.04	0.54^***^	0.03	0.27^***^	0.04	0.44^***^	0.04	0.36^***^	0.04
H_LCA					0.21^***^	0.04				
H_PCR							0.25^***^	0.03		
F_LCA × H_PCR							0.27^***^	0.04		
H_ESI									0.39^***^	0.03
F_LCA × H_ESI									−0.12^***^	0.03
VIF	1.00–1.13		1.00–1.14		1.15–1.49		1.11–1.14		1.01–1.14	
F	30.32^***^		48.56^***^		29.95^***^		62.39^***^		83.32^***^	
*R* ^2^	0.23		0.33		0.28		0.49		0.56	

H_HLCB, Early adolescents’ habitual low-carbon consumption behavior; F_LCA, Parental low-carbon concerns; H_LCA, Early adolescents’ low-carbon concerns; H_PCR, Parent–child relationship; H_ESI, Environmental self-identity, VIF, Variance inflation factor. ^*^p < 0.05, ^**^p < 0.01, ^***^p < 0.001.

**Table 7:** Regression results for the mediation and moderated mediation models (Equations 6–8).

Variables	Equation 6		Equation 7		Equation 8	
	H_PLCB		H_PLCB		H_LCA	
	*B*	*SE*	*B*	*SE*	*B*	*SE*
Constant	2.39^***^	0.18	1.96^***^	0.20	3.89^***^	0.05
Parent’s gender	0.02	0.07	0.03	0.06	−0.01	0.01
Disposable household income	0.01	0.03	0.01	0.03	0.06^***^	0.01
Parents’ educational Level	−0.02	0.04	−0.01	0.04	0.01	0.03
F_LCA	0.42^***^	0.04	0.28^***^	0.05	0.29^***^	0.03
H_LCA			0.26^***^	0.05		
H_PCR					0.26^***^	0.02
F_LCA × H_PCR					0.12^***^	0.03
H_ESI					0.32^***^	0.03
F_LCA × H_ESI					−0.19^***^	0.03
VIF	1.10–1.14		1.10–1.49		1.02–1.37	
F	26.63^***^		28.53^***^		98.30^***^	
*R* ^2^	0.21		0.27		0.67	

H_PLCB, Early adolescents’ purchasing low-carbon consumption behavior; F_LCA, Parental low-carbon concerns; H_LCA, Early adolescents’ low-carbon concerns; H_PCR, Parent–child relationship; H_ESI, Environmental self-identity; VIF, Variance inflation factor. ^*^p < 0.05, ^**^p < 0.01, ^***^p < 0.001.

Hypothesis 1 posits that parental low-carbon concern exerts an indirect effect on early adolescents’ low-carbon consumption behavior through early adolescents’ own low-carbon concern. Because low-carbon consumption comprises habitual and purchasing dimensions (Mi et al., 2019; Cheng et al., 2023), the proposed mediation and moderated mediation models were estimated separately for HLCB and PLCB. The analyses were intended to assess whether the hypothesized associations were evident for both behavioral outcomes, rather than to test a directional difference between them.

As presented in Tables 6, 7, parental low-carbon concern was significantly and positively associated with early adolescents’ low-carbon concern (Eq. 2, *B* = 0.54, *p* < 0.001) and early adolescents’ HLCB (Eq. 1, *B* = 0.38, *p* < 0.001), after controlling for parental education, gender, and household disposable income. Furthermore, the association between early adolescents’ low-carbon concern and their HLCB remained significant (Eq. 3, *B* = 0.21, *p* < 0.001). The mediation analysis revealed a significant indirect effect of early adolescents’ low-carbon concerns in the relationship between parental low-carbon concerns and early adolescents’ habitual low-carbon consumption behavior (HLCB) (effect = 0.11, 95% CI = [0.05, 0.19]), accounting for 28.95% of the total effect. With respect to purchasing low-carbon consumption behavior (PLCB), parental low-carbon concern was positively associated with early adolescents’ PLCB (Eq. 6, *B* = 0.42, *p* < 0.001). Additionally, early adolescents’ low-carbon concern was significantly related to their PLCB (Eq. 7, *B* = 0.26, *p* < 0.001). Mediation analysis further revealed a significant indirect effect of early adolescents’ low-carbon concern in the relationship between parental low-carbon concern and adolescents’ PLCB (indirect effect = 0.15, 95% CI = [0.08, 0.21]), accounting for 35.71% of the total effect. These findings provide full support for Hypothesis 1, underscoring the mediating role of early adolescents’ concern in the intergenerational transmission of low-carbon consumption behaviors.

**Figure 2:**
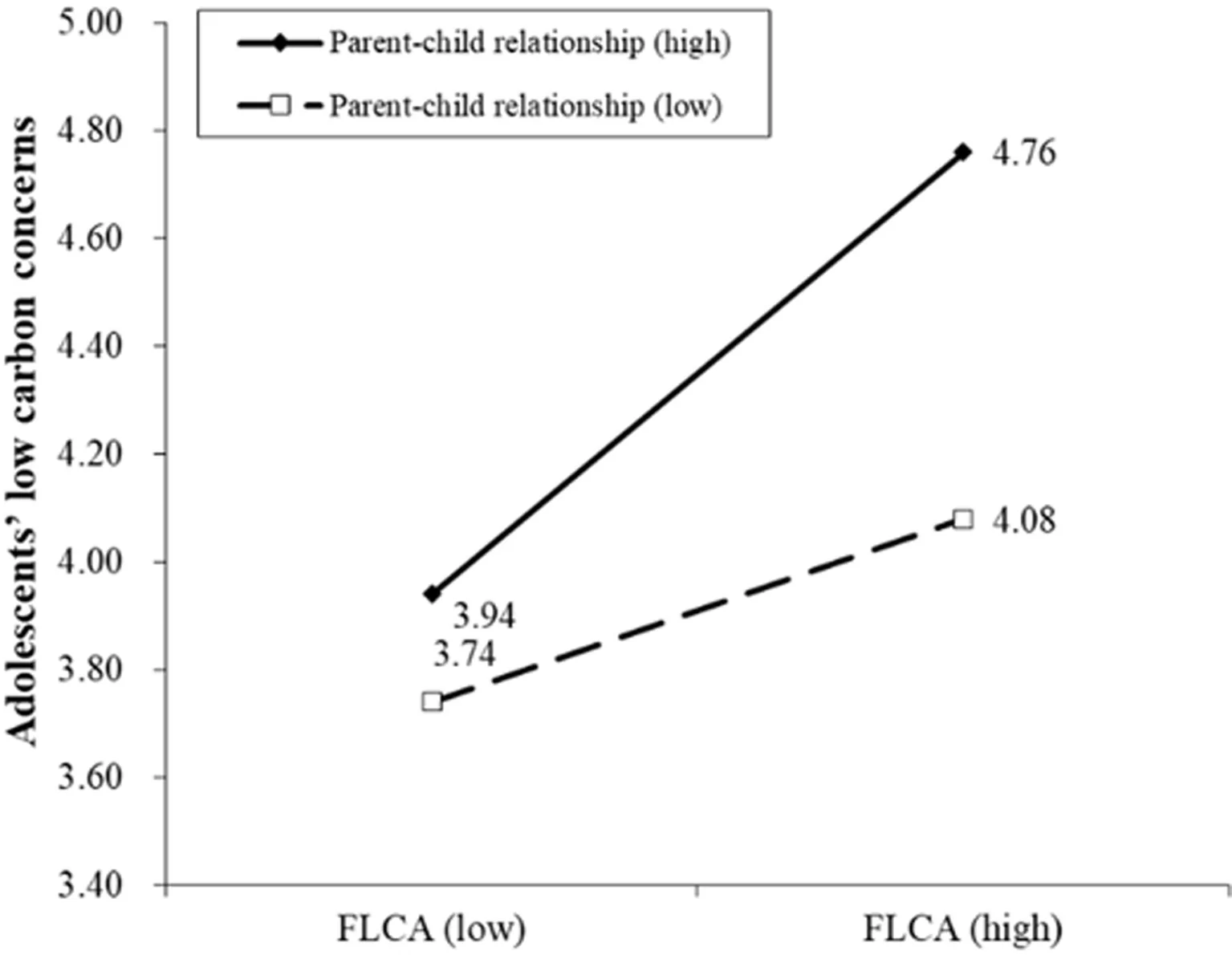
Moderating effect of adolescent-perceived parent–child relationship quality on the association between parental and early adolescents’ low-carbon concern.

To further examine the moderated mediation effect, we employed PROCESS Model 7 (Preacher et al., 2007; Hayes, 2017), specifying early adolescents’ low-carbon concern as the mediating variable and parent–child relationships as the moderator. As shown in Table 6, the interaction between parental low-carbon concern and the quality of the parent–child relationship was statistically significant (Eq. 4, *B* = 0.27, *p* < 0.001). Further simple slope analyses revealed that parental low-carbon concern was positively associated with early adolescents’ low-carbon concern at both levels of parent–child relationship quality. The association was weaker when the parent–child relationship was relatively distant (*B_simple_* = 0.18, 95% CI = [0.06, 0.29]) and stronger when the relationship was close (*B_simple_* = 0.71, 95% CI = [0.62, 0.80]). This pattern is consistent with the positive interaction effect. This moderating effect is illustrated in Figure 2 and is consistent with a stronger positive association between parental and adolescent low-carbon concern under conditions of greater relational closeness.

This study systematically examined the mediating role of early adolescents’ low-carbon concern in the relationship between parental low-carbon concern and two types of low-carbon consumption behaviors—habitual (HLCB) and purchasing (PLCB)—through a moderated mediation model, with particular attention to the moderating effect of the parent–child relationship. Regarding early adolescents’ HLCB, the results indicated a significant parent–child relationship–dependent mediation. Specifically, the indirect effect was stronger and statistically significant when the parent–child relationship was close (*indirect effect* = 0.14, 95% CI = [0.06, 0.24]). By contrast, the indirect effect was weaker and not statistically significant when the relationship was distant (*indirect effect* = 0.04, 95% CI = [−0.01, 0.08]). This conditional indirect effect was supported by the index of moderated mediation (index = 0.06, 95% CI = [0.02, 0.11]).

Similarly, for PLCB, the indirect effect was both stronger and statistically significant when the parent–child relationship was close (*indirect effect* = 0.19, 95% CI = [0.11, 0.28]). In contrast, the mediating effect was not significant when the relationship was distant (*indirect effect* = 0.05, 95% CI = [−0.01, 0.09]). The index of moderated mediation was also significant (*index* = 0.07, 95% CI = [0.04, 0.13]), further supporting the existence of a conditional indirect effect. These findings support H2 for both HLCB and PLCB. Because the difference between the two conditional indirect effects was not directly tested, the apparent variation across outcomes should be interpreted descriptively.

**Figure 3:**
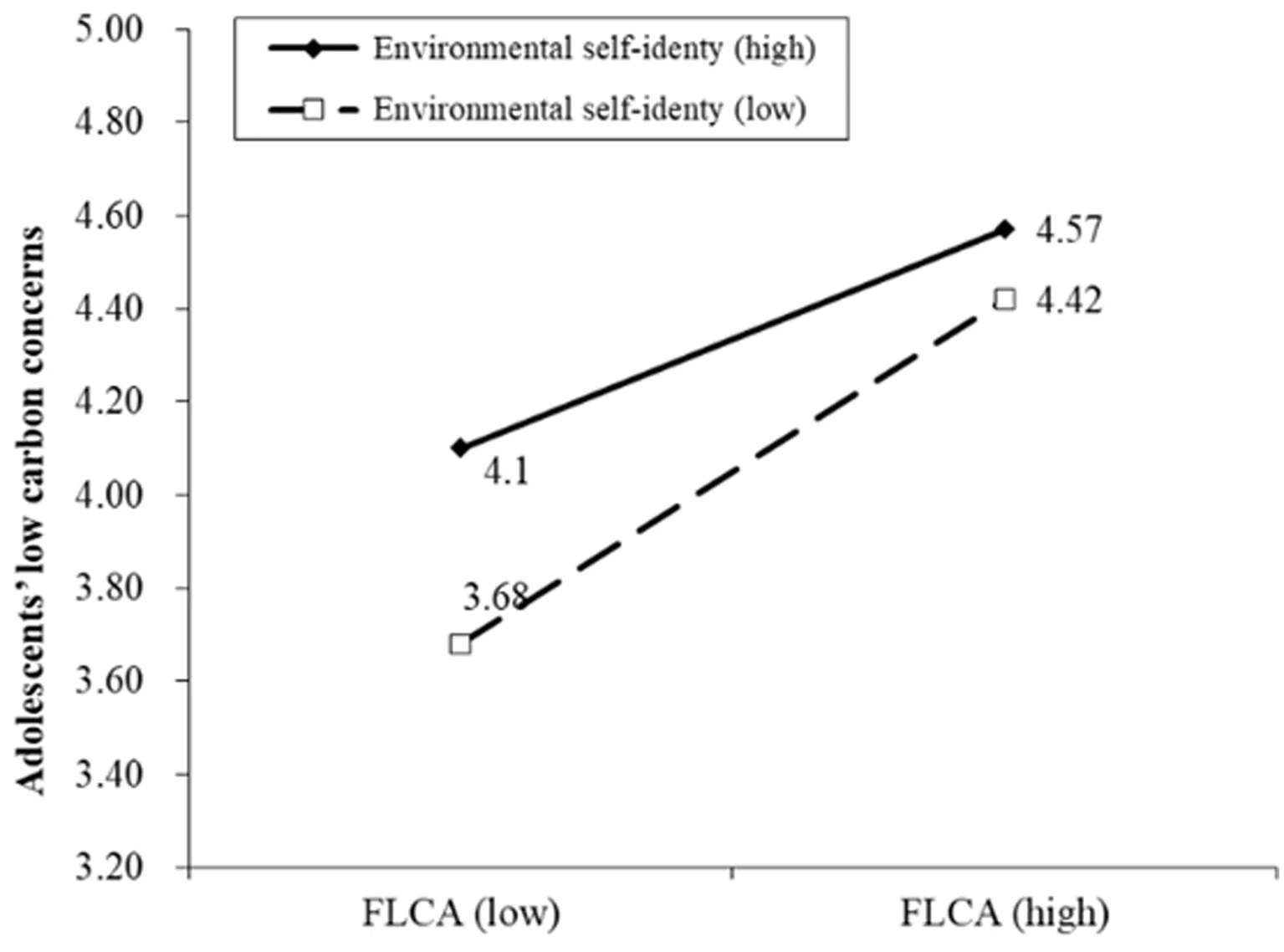
Moderating effect of early adolescents’ environmental self-identity on the association between parental and early adolescents’ low-carbon concern.

Environmental self-identity moderated the first-stage path from parental low-carbon concern to early adolescents’ low-carbon concern. As shown in Table 6, the interaction between parental low-carbon concern and early adolescents’ environmental self-identity was statistically significant (Eq. 5, *B* = −0.12, *p* < 0.001). A simple slope analysis further showed that parental low-carbon concern was more strongly associated with early adolescents’ low-carbon concern when environmental self-identity was low (*B_Simple_* = 0.45, 95% CI = [0.37, 0.51]). The association remained statistically significant but was weaker when environmental self-identity was high (*B_simple_* = 0.26, 95% CI = [0.17, 0.36]). This pattern is consistent with the negative interaction effect. Figure 3 provides a graphical illustration of this interaction effect.

This study further examined the mediating role of early adolescents’ low-carbon concern in the relationship between parental low-carbon concern and early adolescents’ HLCB, across different levels of environmental self-identity. The results showed that the indirect effect was statistically significant but modest when early adolescents reported high levels of environmental self-identity (*indirect effect* = 0.05, 95% CI = [0.02, 0.10]). In contrast, the mediating effect was stronger and statistically significant when environmental self-identity was low (*indirect effect* = 0.09, 95% CI = [0.04, 0.15]). Furthermore, the index of moderated mediation was also significant (*index* = −0.02, 95% CI = [−0.05, −0.01]), confirming the presence of a conditional indirect effect. Similarly, for purchasing low-carbon consumption behavior (PLCB), the indirect effect was statistically significant but smaller when early adolescents reported higher levels of environmental self-identity (*indirect effect* = 0.07, 95% CI = [0.03, 0.12]). In contrast, the mediating effect was stronger and statistically significant under conditions of low environmental self-identity (*indirect effect* = 0.12, 95% CI = [0.07, 0.18]). Moreover, the index of moderated mediation was significant (*index* = −0.03, 95% CI = [−0.06, −0.01]), providing further evidence for a conditional indirect effect. These findings highlight the crucial moderating role of early adolescents’ environmental self-identity in the intergenerational transmission of low-carbon consumption behavior. However, contrary to our expectations, the direction of the moderation effect was inconsistent with our hypothesis, and therefore Hypothesis 3 was not supported.

To test the complete conditional process model, we estimated PROCESS Model 9 separately for HLCB and PLCB. Parent–child relationship quality and environmental self-identity were simultaneously specified as moderators of the association between parental and adolescent low-carbon concerns. The interaction involving parent–child relationship quality was positive and significant (Eq. 8, *B* = 0.12, *p* < 0.001), whereas the interaction involving environmental self-identity was negative and significant (Eq. 8, *B* = −0.19, *p* < 0.001).

The conditional indirect effects followed the same pattern for both behavioral outcomes. They increased as the parent–child relationship became closer but declined as environmental self-identity strengthened. For HLCB, the indirect effect ranged from 0.01 (95% CI = [−0.02, 0.02]) under high environmental self-identity and a weak parent–child relationship to 0.11 (95% CI = [0.05, 0.19]) under low environmental self-identity and a close relationship. For PLCB, the indirect effect ranged from 0.01 (95% CI = [−0.03, 0.03]) to 0.14 (95% CI = [0.08, 0.22]) under the corresponding conditions. For both outcomes, the indirect effect was nonsignificant only when environmental self-identity was high and the parent–child relationship was weak.

The indices of partial moderated mediation were significant for both moderators and both behavioral outcomes. For HLCB, the indices of partial moderated mediation were significant for parent–child relationship quality (index = 0.02, 95% CI = [0.01, 0.05]) and environmental self-identity (index = −0.04, 95% CI = [−0.07, −0.02]). Similarly, for PLCB, the indices of partial moderated mediation were significant for parent–child relationship quality (*index* = 0.03, 95% CI = [0.01, 0.06]), and environmental self-identity (*index =* −0.05, 95% CI = [−0.08, −0.02]). These results support H2. The significant effect of environmental self-identity was opposite to the direction predicted in H3. H3 was therefore not supported.

## 6 Discussion

The issue of low-carbon consumption has gained increasing scholarly attention across the domains of consumer ethics and environmental sustainability (Jiang et al., 2019; Mi et al., 2019; Singh et al., 2020; Ding et al., 2025). However, existing research predominantly centers on adult populations, overlooking the formative role of early adolescents in shaping long-term low-carbon consumption patterns. Given that the United Nations Sustainable Development Goals (SDGs) identify children and early adolescents as critical agents of environmental change (UNESCO, 2017), examining low-carbon behaviors during this critical life stage is essential for designing early interventions and educational strategies aimed at long-term sustainability. As future consumers and societal contributors, early adolescents’ low-carbon behaviors have profound implications for sustainable development trajectories (Kong and Jia, 2023). This study addresses this gap by investigating the role of parental low-carbon concerns in shaping early adolescents’ low-carbon consumption behaviors. Drawing on ecological socialization theory (Gentina and Muratore, 2012), we examined whether parental low-carbon concern is associated with adolescents’ own concern, which in turn is linked to their habitual and purchasing low-carbon behaviors.

The findings revealed three major insights. First, early adolescents’ low-carbon concerns were positively associated with parental concerns and, in turn, with their consumption behaviors. Second, parent–child relationship strengthened the indirect association for both habitual and purchasing low-carbon consumption.

Contrary to H3, environmental self-identity weakened rather than strengthened the association between parental and adolescent low-carbon concern. This result should not be interpreted as evidence that environmental self-identity inhibits early adolescents’ low-carbon development. Instead, it indicates that parental concern was less strongly associated with early adolescents’ concern when adolescents reported a stronger environmental self-identity.

Several nonexclusive explanations are possible. First, environmental self-identity and low-carbon concern are conceptually related. Early adolescents with a strong environmental identity may already report relatively high levels of low-carbon concern, leaving less variation to be associated with parental concern. This pattern may therefore reflect conceptual overlap, restricted variance, or a saturation effect. Second, suppression or model dependence cannot be completely excluded. However, the negative interaction appeared in both the separate and simultaneous moderation models, and the VIF values ranged from 1.00 to 1.49, suggesting that severe multicollinearity was unlikely to explain the result.

Third, alternative model specifications are plausible. Environmental self-identity may operate as an antecedent of low-carbon concern or may strengthen the association between concern and behavior, rather than moderating the transmission of concern from parents to early adolescents. The current design cannot distinguish among these possibilities. Finally, a developmental explanation remains possible. Environmental self-identity represents an internalized self-definition that can guide pro-environmental judgments and behavior (Tajfel, 1986; van der Werff et al., 2013). As this identity becomes more established, early adolescents may attach less incremental importance to parental cues and rely more on their own values when forming low-carbon concerns. Conversely, early adolescents with a weaker environmental identity may depend more heavily on parental modeling, communication, and reinforcement (Meeusen, 2014; Gentina and Muratore, 2012). These explanations were not directly tested and should therefore be regarded as exploratory. Longitudinal and experimental studies, together with comparisons of alternative structural models, are needed to determine whether the observed pattern reflects construct overlap, model specification, or adolescents’ increasing identity-based autonomy.

Collectively, these findings suggest that parental low-carbon concern, parent–child relationship quality, and early adolescents’ environmental self-identity jointly condition early adolescents’ orientation toward sustainable consumption. Our study contributes both theoretically and practically to the understanding of environmental behavior formation in youth, emphasizing the importance of nuanced, context-specific approaches in promoting ethical consumption.

### 6.1 Theoretical contributions

This study makes three focused contributions to research on family environmental socialization and low-carbon consumption. First, it extends ecological socialization research to early adolescents’ consumption-related low-carbon concern. Prior studies have documented intergenerational associations in environmental values and behaviors, including energy conservation, recycling, and green purchasing (Matthies et al., 2012; Hansen and Jacobsen, 2020; Wallis and Klöckner, 2020; Aruta and Paceño, 2022; Gong et al., 2022). Research on low-carbon consumption, however, has concentrated largely on adult consumers and individual-level determinants (Ding et al., 2018; Ren et al., 2024; Zhang et al., 2022). We connect these two streams by examining how parental concern about consumption-related carbon emissions is associated with early adolescents’ own concern and consumption behavior. The contribution therefore lies in applying ecological socialization theory to a carbon-specific consumption context and a formative developmental stage, rather than in establishing parental environmental influence for the first time.

Second, the study specifies early adolescents’ low-carbon concern as a psychological pathway in the intergenerational process. Prior research has largely documented parent–child similarities in environmental attitudes, values, and behaviors (Hansen and Jacobsen, 2020; Wallis and Klöckner, 2020), while providing less insight into how parental environmental orientations become associated with adolescents’ consumption practices. Our findings show that early adolescents’ low-carbon concern helps explain the association between parental concern and both habitual and purchasing low-carbon consumption. Examining these outcomes separately demonstrates that the proposed process applies to both routine practices and deliberate purchasing decisions.

Third, the study incorporates relational and identity-based conditions into the same conditional process model. Parent–child relationship captures the relational climate in which parental messages are interpreted and internalized. A closer relationship strengthened the association between parental and adolescent low-carbon concerns, consistent with the view that trust and emotional closeness enhance receptiveness to parental guidance (Koerner and Fitzpatrick, 2002; Gong et al., 2022). Environmental self-identity, however, produced an unexpected negative moderation pattern and did not support the original directional hypothesis. This finding raises the possibility that an established environmental identity reduces the incremental relevance of parental cues. Nevertheless, this interpretation remains exploratory, and alternative explanations involving conceptual overlap, suppression, restricted variance, and model specification cannot be excluded. Longitudinal and experimental research is needed to clarify the mechanism underlying this pattern.

### 6.2 Practical implications

Cultivating low-carbon awareness and behaviors among adolescents is strategically vital for long-term emission reduction and sustainable development (Bai and Liu, 2013; Tian et al., 2025). Yet, this issue has received limited attention from Chinese policymakers and businesses. The findings provide several cautious implications for promoting early adolescents’ low-carbon consumption. First, adolescence is an important period for developing environmental awareness and consumption habits (Bai and Liu, 2013; Tian et al., 2025; UNESCO, 2017). The indirect association through early adolescents’ low-carbon concern suggests that family-based initiatives may help parents communicate and demonstrate the carbon consequences of everyday consumption. Schools, communities, and public authorities could provide practical resources that encourage families to participate in energy conservation, waste reduction, recycling, and sustainable purchasing activities, consistent with the emphasis of education for sustainable development on family and youth engagement (Tian et al., 2023).

Second, adolescent-perceived parent–child relationship quality conditioned the association between parental and adolescent low-carbon concern. This finding suggests that early adolescents may be more receptive to parental environmental messages when they perceive the relationship as close and supportive (Gong et al., 2022; Cheng et al., 2023). Shared activities, such as family energy-saving challenges, recycling programs, and low-carbon learning workshops, may provide opportunities for environmental communication and participation. When perceived relational closeness is weaker, schools and communities may offer complementary sources of environmental learning and social support (Meeusen, 2014; Mohamed et al., 2023). These activities should nevertheless be regarded as potential applications rather than interventions whose effectiveness has been established by the present study.

Finally, the unexpected moderation pattern cautions against assuming that stronger environmental self-identity necessarily increases early adolescents’ receptiveness to parent-centered messages. Because the mechanism underlying this result remains uncertain, differentiated communication strategies should be empirically tested before being translated into interventions. For early adolescents with stronger environmental self-identity, messages emphasizing personal agency, moral responsibility, and self-directed environmental goals may be more appropriate than appeals centered primarily on parental guidance (Fu et al., 2021). Adolescents with weaker environmental self-identity may remain more responsive to parental modeling, communication, and reinforcement. Community recognition programs, such as low-carbon family initiatives, and credible green labeling may also help make sustainable behavior more visible and personally meaningful (Lukova, 2024; Di et al., 2024). However, these possibilities extend beyond the present findings and require direct experimental evaluation.

### 6.3 Limitations and future research

This study is not without limitations, which should be acknowledged as they offer important avenues for future research. First, the generalizability of the findings is limited by the study’s cultural and developmental scope. The study was conducted exclusively in China, where collectivism, filial piety, and hierarchical family roles may strengthen the role of parents in adolescents’ socialization and consumption decisions (Xu et al., 2005; Gong et al., 2022). Because cultural values shape both the content and transmission of environmental beliefs, the observed associations may differ in more individualistic and autonomy-oriented contexts (Gentina and Singh, 2015; Jiang et al., 2019; Pearce et al., 2020; Wang J. et al., 2022). In addition, the sample was concentrated in early adolescence, particularly among those aged 12–13 years. This age homogeneity supports the study’s focus on early family socialization but limits its applicability to older adolescents. Future research should conduct cross-cultural comparisons and recruit age-stratified samples across early, middle, and late adolescence to examine whether the proposed associations vary across cultural and developmental contexts.

Second, although parental low-carbon concern was measured 1 month before the adolescent variables, the observational design does not establish temporal ordering or causality. Unmeasured family, social, or individual factors may partly account for the observed associations. Longitudinal cross-lagged, diary, and experimental studies are therefore needed to assess directionality and examine how low-carbon concerns and behaviors develop over time. In addition, participation was limited to schools willing to cooperate, which may introduce selection bias despite recruiting from multiple regions in China. Future research should use broader sampling and mixed-method designs, including longitudinal tracking, field experiments, and ecological momentary assessment, to strengthen causal inference and external validity.

Third, although this study identifies adolescents’ low-carbon concern as a mediating pathway consistent with ecological socialization theory (Gentina and Muratore, 2012; Gentina and Singh, 2015), it captures only part of the intergenerational transmission process. Parents may shape adolescents’ consumption through modeling, reinforcement, communication, and shared interaction (Moschis, 1985). Future research should therefore examine additional mechanisms, such as value internalization, moral reasoning, and emotional attachment to nature. Other contextual and individual conditions, including peer influence, media exposure, environmental policy awareness, and cognitive maturity, may also interact with parental influence in shaping low-carbon behavior (Ding et al., 2018; Zhang et al., 2022). The unexpected negative moderation by environmental self-identity also warrants caution. Although the interaction appeared in both the separate and simultaneous models, the present study cannot determine whether it reflects conceptual overlap, suppression, restricted variance, alternative model specification, or adolescents’ increasing autonomy. Future studies should compare competing structural models and employ longitudinal or experimental designs to distinguish these explanations.

Fourth, although parental low-carbon concern was reported by parents and collected 1 month before the adolescent survey, adolescents self-reported their own low-carbon concern, environmental self-identity, parent–child relationship quality, and both behavioral outcomes. Parent–child relationship quality therefore reflects adolescents’ perceptions rather than parents’ perceptions or a shared relational characteristic. Although such perceptions are relevant to adolescents’ receptiveness to parental influence, associations among the adolescent-reported variables may partly reflect common-source variance, consistency motives, or social desirability. The Harman and ULCMF tests indicated no dominant method factor, but these diagnostics cannot fully eliminate this possibility. The findings should therefore be interpreted as conditional associations. Future research should incorporate parent reports, other informants, objective behavioral measures, and temporally separated assessments.

Finally, although the matched parent-adolescent multi-informant design separated the parental predictor from the adolescent-reported variables, the analysis did not fully model dyadic interdependence. Parallel predictors and outcomes were not collected from both dyad members, and reciprocal parent-adolescent effects were beyond the scope of the study. Future research should collect comparable measures from both parents and adolescents and apply APIM or dyadic SEM to examine actor, partner, and bidirectional socialization effects. Such research could also investigate reverse socialization, whereby adolescents influence their parents’ environmental attitudes and behaviors (Singh et al., 2020; Kong and Jia, 2023), as well as how adolescents challenge or reinforce sustainable and unsustainable family norms.

## Data Availability

The original contributions presented in the study are included in the article/supplementary material, further inquiries can be directed to the corresponding author.
